# Agnostic detection of genomic alterations by holistic DNA structural interrogation

**DOI:** 10.1371/journal.pone.0208054

**Published:** 2018-11-29

**Authors:** Ryan K. Shultzaberger, Rachel E. Abrams, Challise J. Sullivan, Anthony D. Schmitt, Thomas W. J. Thompson, John Dresios

**Affiliations:** 1 Leidos, Inc., San Diego, CA, United States of America; 2 Arima Genomics Inc., San Diego, CA, United States of America; Hirosaki University Graduate School of Medicine, JAPAN

## Abstract

There is an established relationship between primary DNA sequence, secondary and tertiary chromatin structure, and transcriptional activity, suggesting that observed differences in one of these properties may reflect changes in the others. Here, we exploit these relationships to show that variations in DNA structure can be used to identify a wide range of genomic alterations in mammalian samples. In this proof-of-concept study we characterized and compared genome-wide histone occupancy by ChIP-Seq, DNA accessibility by ATAC-Seq, and chromosomal conformation by Hi-C for five CRISPR/Cas9-modified mammalian cell lines and their unmodified parent strains, as well as in one modified tissue sample and its parent strain. The results showed that the impact of genomic alterations on each of the levels of DNA organization varied depending on mutation type (insertion or deletion), size, and genomic location. The largest genomic alterations we identified included chromosomal rearrangements and deletions (greater than 200 Kb) in four of the modified cell lines, which can be difficult to identify by standard whole genome sequencing analysis. This multi-level DNA organizational analysis provides a sensitive approach for identifying a wide range of genomic and epigenomic perturbations that can be utilized for biomedical and biosecurity applications.

## Introduction

DNA does not exist in the cell as a naked, linear molecule, but rather is compacted into a highly organized three-dimensional structure that exerts genome-wide influence over gene expression levels [1, 2]. The first level of compaction is achieved through the regular wrapping of DNA around histone octamers, forming a compact chain of nucleosomes that restricts access of the transcriptional machinery to the associated DNA (secondary DNA structure) [3, 4]. The DNA accessibility, and subsequently transcriptional activity, of a given locus can vary between cell types or physiological conditions depending on the presence of specific DNA-binding proteins that can covalently modify histone proteins and disrupt nucleosome stability [5, 6]. These strings of nucleosomes are further organized into a higher level tertiary structure consisting of loops that are formed by protein-mediated promoter-enhancer interactions, which in turn are contained within larger topological associating domains (TADs) [7, 8]. TADs promote looping by providing a local structure to facilitate promoter-enhancer interactions. TAD boundaries are thought to be established by the sequence-specific DNA-binding protein CTCF and to be invariant between tissue types [8, 9]. TADs are organized into larger, functionally related compartments [10, 8]. As all of these levels of organization are affected by the underlying DNA sequence, measurable variations in these structures may provide new power in mutation detection as well as allow for the characterization of the effect of environmental exposure on the epigenomic landscape, as we previously suggested [11].

Genomic and epigenomic perturbations can result from exposure to a variety of environmental factors [12–14], physiological stressors [15–17] as well as direct manipulation of the host organism (e.g., genome editing) [11, 18–20]. Genome editing mediated by CRISPR/Cas9 has enabled the manipulation of nearly any organism and has been widely utilized in basic research through the modification of cell lines and model organisms [18]. In addition, its effective delivery of targeted genomic alterations, direct or indirect, has also been reported [21–24]. In this regard, CRISPR-Cas9 modified eukaryotes can serve as examples for developing comprehensive methods toward agnostic detection of target and secondary genomic and epigenomic modifications. Existing approaches have shown power in identifying secondary mutations in CRISPR/Cas9-modified samples when the sequence of the utilized guide RNA (gRNA) is known [25–28], but their search is often restricted to genomic regions that are accessible to Cas9 *in vitro* or have homology to the gRNA sequence, thus resulting in a partial capture of secondary mutation sites. None of these methods can be applied to identify genome editing sites when the gRNA sequence is not known as required for agnostic detection of genome editing for biosecurity. Whole genome sequencing provides greater coverage of the genome than gRNA-centric approaches, but some mutation types (chromosomal rearrangements, large insertions and deletions) can be challenging to identify from these data [29].

**Fig 1:**
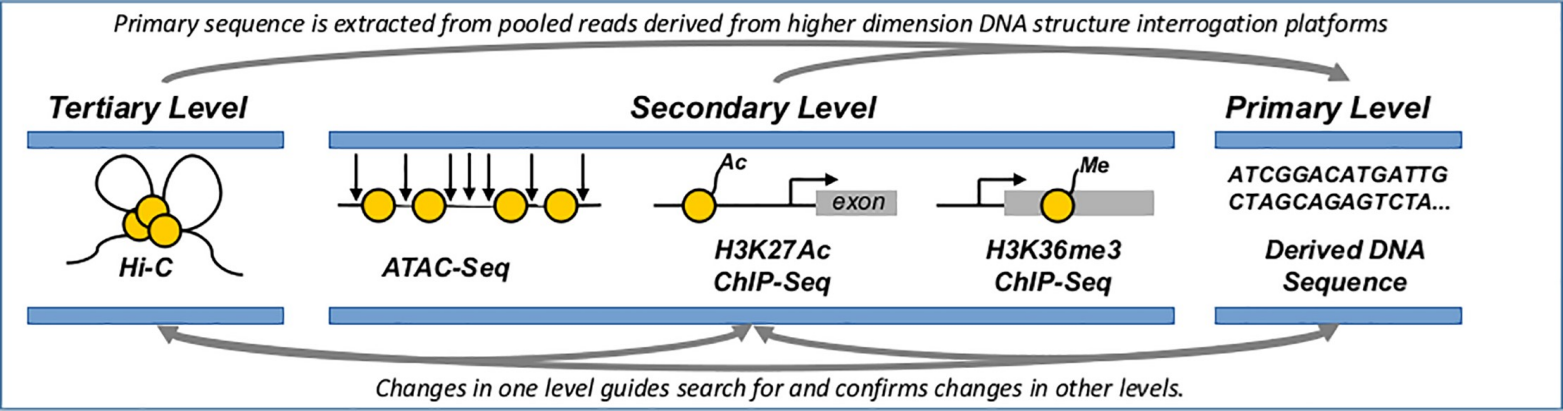
Holistic capture of DNA organizational levels. We use three different NGS-based assays that capture DNA structural levels genome-wide. These are: **Hi-C**, which identifies regions of the genome that are close to each other in 3D space (Tertiary Structure), and has shown power in identifying local and global structural changes, as well as large deletions and chromosomal rearrangements. **ATAC-Seq**, which identifies regions of the genome that are accessible to protein binding, like Cas9, providing enriched coverage in potential sites of off-target modification. **ChIP-Seq** on histone variants, which characterizes histone modification and occupancy indicative of variations in transcriptional state. Sequencing data derived from aforementioned platforms can also be combined to generate nearly complete primary sequence coverage.

To test the detection of genomic alterations through variations in DNA structures, three complementary next generation sequencing (NGS)-based assays were selected, which, collectively, provide a comprehensive interrogation of the impact of CRISPR/Cas9-induced mutations on the structural organization of mammalian genomes (Fig 1). These assays are: 1) Chromatin Immunoprecipitation followed by Sequencing (ChIP-seq), which captures secondary structure in the form of DNA-protein interactions by crosslinking proteins to the DNA, fragmenting the DNA, immunoprecipitating out a specific histone variant, and sequencing the associated DNA-fragments [30–31]; 2) Assay for Transposase-Accessible Chromatin using Sequencing (ATAC-Seq), which captures secondary structure in the form of DNA accessibility by adding a hyper-active transposase that cuts accessible regions of the genome and ligates an adapter to the cut region for direct NGS-based sequencing [32]; and 3) Chromosome Conformation Capture (Hi-C), which captures tertiary structure in the form of inter- and intra-chromosomal associations by treating crosslinked chromatin with a restriction enzyme, labeling the digested ends with an affinity purification tag, ligating proximal loci, followed by affinity-enrichment and sequencing of the ligation junctions [10]. Because these technologies are all NGS-based, primary sequence information is also captured by simply combining raw sequencing reads from all platforms. Here, we show that identifying statistically significant changes in each of these structural levels can guide the identification of genomic alterations across the genome and provides increased confidence in called mutations through multiple, independent validations.

## Results

### Multi-dimensional DNA structural data analysis

We obtained 5 mammalian cell lines and a mouse tissue sample where a targeted deletion was induced by CRISPR/Cas9, and the parent strains from which these mutants were derived (Table 1). The mutant samples consisted of three edited human cell lines (BRD4, TOP2B, SMARCA4) derived from a single HAP1 parent strain, two edited mouse embryonic stem cell lines (Sox2, SE15) derived from a single F123 parent strain [33–34], and one edited mouse liver tissue sample (Kcnc3) derived from a C57BL/6NJ parent strain. All samples are named based on the gene or regulatory element that contained the targeted deletion. Sox2 and SE15 are deletions to super-enhancers (SEs); all other samples contain deletions in exons. Samples were selected covering a range of deletion sizes (4 bp—40 Kb) in order to probe the resolution of mutation detection for each structural feature. High quality ChIP-Seq, ATAC-Seq, and Hi-C data were generated for two biological replicates of all modified samples and their reference backgrounds (S8 Table). ChIP-Seq was performed against the histone variants H3K27Ac and H3K36me3 which mark transcriptionally active enhancers and exons respectively [35–36]. There were at least 17 million uniquely mapped ATAC-Seq and ChIP-Seq reads for each experiment and 450 million reads for each Hi-C experiment. For the HAP1-derived samples, these combined data covered approximately 85% of the genome at 1X coverage, 70% at 5X coverage, and 60% at 10X (S1 Fig). ChIP-Seq and ATAC-Seq peak densities were highly correlated between samples of the same background for each data type (S2 Fig), suggesting that biological and technical variation between samples are low.

**Table 1:** Sample information.

Sample	Species	Background	Del Size (bp)	Location	Gene Description
BRD4	Human	HAP1 Cell Line	4	Exonic	Histone binding protein that helps maintain chromatin structure during mitosis
TOP2B	Human	HAP1 Cell Line	19	Exonic	DNA Topoisomerase subunit that helps regulate topological state of DNA
SMARCA4	Human	HAP1 Cell Line	41	Exonic	Transcriptional regulator that functions through chromatin remodeling
Kcnc3	Mouse	C57BL/6NJ Liver Tissue	1,182	Exonic	Voltage-dependent potassium channel
Sox2	Mouse	F123 ES Cell Line	12,984	Intergenic	Super-enhancer that controls expression of the Sox2 gene that regulates pluripotency
SE15	Mouse	F123 ES Cell Line	40,744	Intergenic	Super-enhancer that regulates at least 100 loci

**Fig 2:**
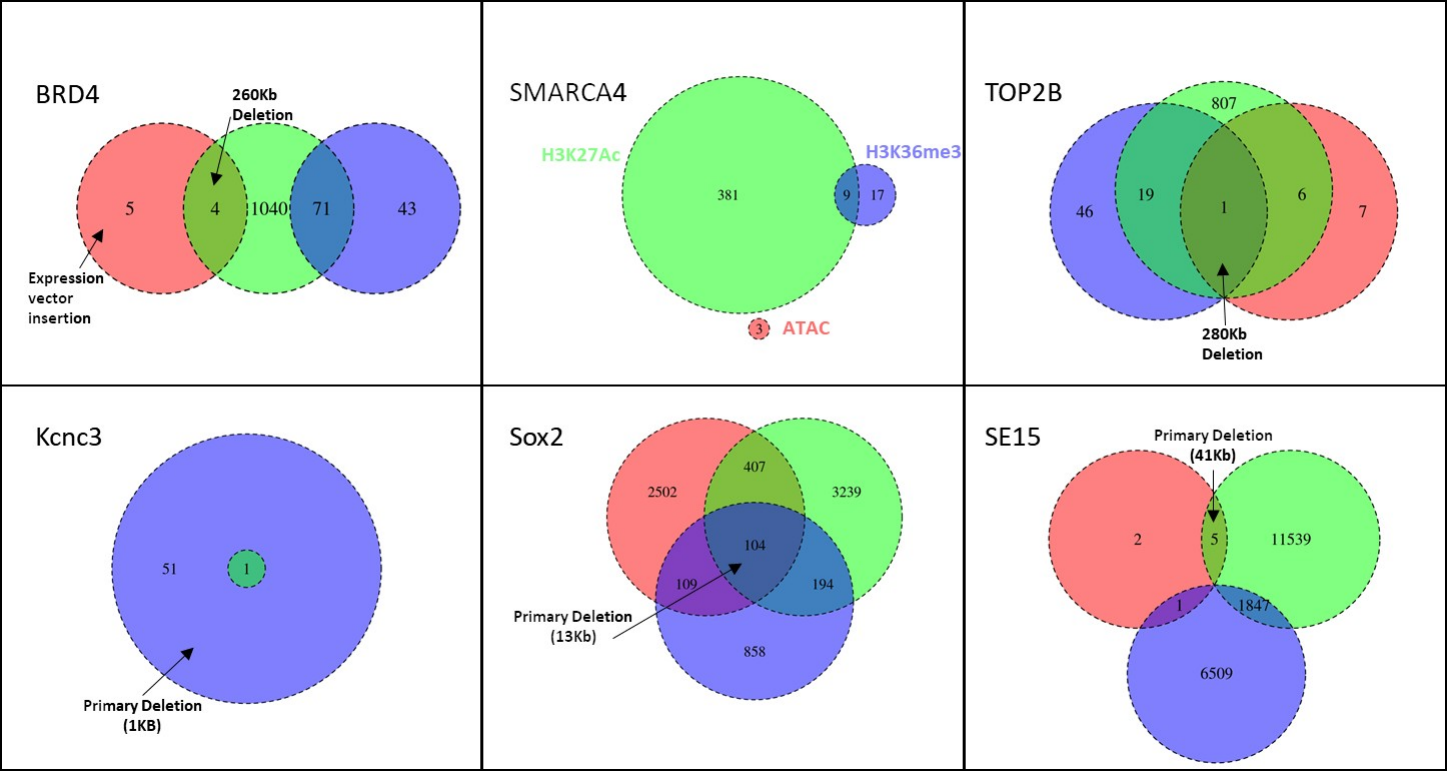
Intersection of differential ATAC- and ChIP-Seq peaks. Venn Diagrams show the overlap between differentially accessible/occupied ATAC-Seq (Red), H3K27Ac ChIP-Seq (Green), and H3K36me3 ChIP-Seq (Blue) peaks for all six mutants relative to their parent strain (see Materials and Methods). Peaks were counted as overlapping if they were within 5Kb of a peak of a different platform (see Materials and Methods). Arrows designate which differential analyses identified the associated mutation. The large deletions and insertion indicated in the BRD4 and TOP2B samples are new mutations identified in our analysis and further characterized in S5 Fig and S6 Fig. Information for each called differential peak is reported in S1–S6 Tables.

We performed differential ATAC-Seq, ChIP-Seq, and Hi-C analysis on all modified samples relative to their reference background strain. For the ATAC- and ChIP-Seq data, differential peak analysis was performed using DESeq2 [37], where regions that have significantly different DNA accessibility or histone occupancy between the CRISPR/Cas9-modified sample and the reference background are identified. A summary of the number of differential peaks for each assay is given in Fig 2. On average, there was 6.2 times more significantly different H3K27Ac ChIP-Seq peaks than H3K36me3 peaks, suggesting that the H3K27Ac modification is more transient than H3K36me3. Significantly different ATAC-Seq peaks were less common than differential peaks for either of the histone variants, but this is largely explained by differences between these methods in the library preparation and analysis of these data (see Materials and Methods). Fewer than 20 differential ATAC-Seq peaks were observed for all samples except for Sox2 mutant, which had around 3,100 (Fig 2). Sox2 is one of three transcription factors that regulate pluripotency [38], therefore differential regulation of this gene could dramatically impact the transcriptome and the DNA accessibility profile. This result is consistent with a large number of differential H3K27Ac and H3K36me3 ChIP-Seq peaks in the Sox2 mutant sample, which also reflect variations in transcriptional state. The SE15 mutant sample had the greatest number of differential H3K27Ac and H3K36me3 peaks out of all of the strains, but only a small number of differential ATAC-Seq peaks. To better understand how changes in DNA accessibility, H3K27Ac modification, and H3K36me3 modification are related, we determined how often differential peaks in one data type were proximal to (within 5 Kb) differential peaks in the others. The extent of overlap between data types varied between samples, but generally H3K27Ac differential peaks were proximal to ATAC-seq and H3K36me3 peaks more frequently than they were to each other (Fig 2).

We performed differential Hi-C analysis using diffHiC [39] to identify 25 Kb blocks that were differentially associated between the mutant and parent strains (Differential Interaction Analysis), and called peaks that were differentially associated (Differential Peak Analysis, see Materials and Methods). For the HAP1-derived mutants, a few significant changes were called. Differential interaction analysis identified one significantly different region for the BRD4 mutant on Chromosome 11, which is proximal to differential ATAC-Seq and H3K27Ac ChIP-Seq peaks, and five significantly different interactions for the TOP2B mutant contained within the same region on Chromosome 10; these interactions are proximal to differential peaks for all other platforms. The SMARCA4 mutant sample had no significantly differential interactions. Differential peak analysis identified one significantly different peak for the BRD4 mutant that overlapped three differential H3K27Ac peaks, and one and two differential peaks for SMARCA4 and TOP2B mutants, respectively, neither of which were proximal to an ATAC- or ChIP-Seq differential peak. The F123-derived Sox2 mutant had tens of thousands of called differential interactions spread across Chromosomes 7, 8, 13, and 14. Chromosomes 8, 13, and 14 all appear to have undergone inter-chromosomal rearrangements, likely explaining these differences (S3 Fig). Hundreds of called differential peaks were also observed for Chromosomes 8 and 13. The SE15 mutant sample had more than 15,000 called differential interactions, and 140 differential peaks called on Chromosome 15, suggesting intra-chromosomal rearrangement (S4 Fig). No differential interactions or peaks were called for the Kcnc3 mutant sample. Differential analysis results for all platforms are provided in S1–S6 Tables.

### Characterization of targeted deletion sites

**Fig 3:**
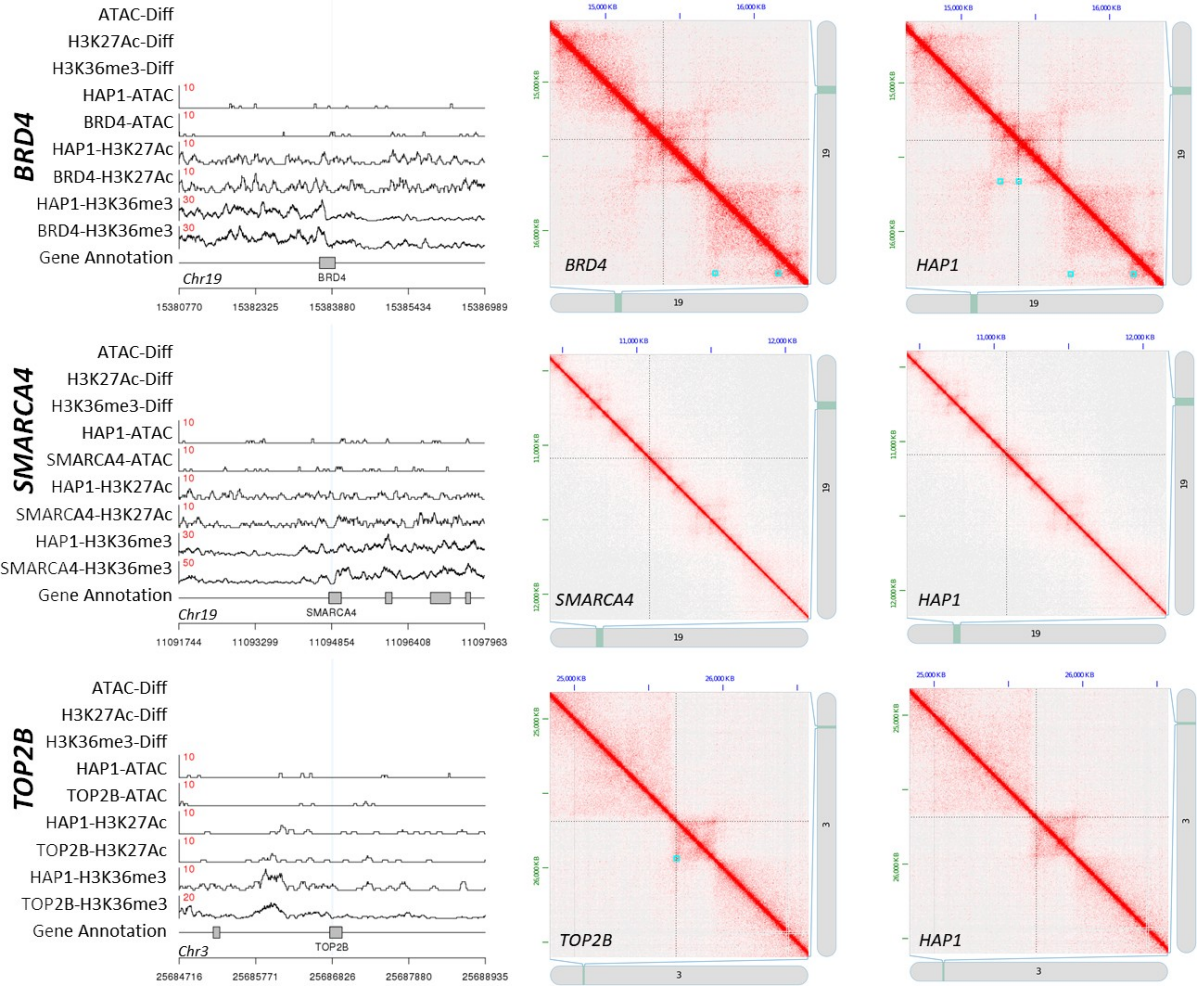
Structural impact of CRISPR/Cas9-targeted mutations in human cell lines. Left three images: Read depth (y-axis) is shown as a function of genomic position (x-axis) for representative ATAC-Seq, H3K27Ac ChIP-Seq, and H3K36me3 ChIP-Seq data for the specified HAP1 cell line at the location of the targeted CRISPR/Cas9 deletions (i.e., HAP1-ATAC is ATAC-Seq sequencing read depth for the HAP1 parent strain). The red number on each trace designates the scale for that trace. Vertical blue lines designate the deletion site. Differentially accessible or occupied regions are marked with red boxes in the top three rows labeled “-Diff". No differential peaks were observed for these deletions. Right six images: Hi-C contact maps show which regions of the genome are proximal to each other. Darker red regions exhibit relatively higher levels of association. Contact maps are shown for the same region in the CRISPR/Cas9 modified strain (left) and the unmodified parent strain (right). The location of the primary deletion is at the intersection of the two dashed lines. Blue boxes designate called loops for each data set. Loops are only marked on one half of the contact map to allow visualization of the corresponding region on the other half.

Our initial analysis focused on the impact of the six known CRISPR/Cas9 target site mutations on all structural levels. We did not observe a significant change in histone occupancy or DNA accessibility at the primary deletion site or its proximity (±5Kb) for the 4 bp BRD4 deletion, the 19 bp TOP2B deletion, or the 41 bp SMARCA4 deletion (Fig 3). The BRD4 and SMARCA4 mutations were both contained within a called H3K36me3 ChIP-Seq peak, but neither mutation significantly affected histone occupancy in these regions. Interestingly, the 4 bp BRD4 deletion did result in the loss of a loop in the encompassing region as well as in a neighboring area that were both called in all other HAP1 strains (Fig 3). The SMARCA4 deletion, which was contained within a loop, had no impact on tertiary structure, and neither did the TOP2B deletion, which was not contained within a loop (Fig 3). All three deletions were identified in the cumulative primary sequencing data from all three platforms by FreeBayes [40] (p ≤ 1e-28).

**Fig 4:**
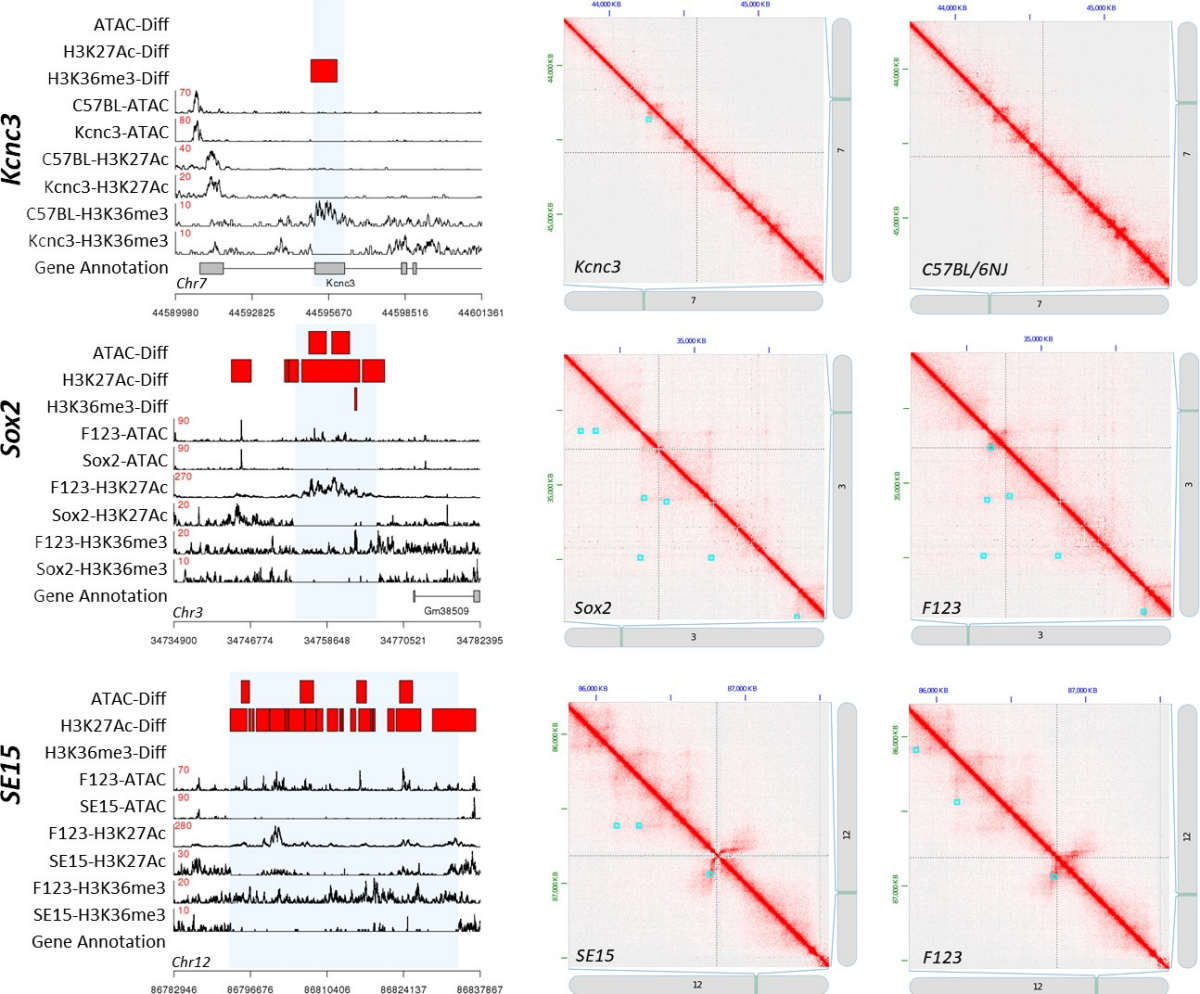
Structural impact of CRISPR/Cas9-targeted mutations in mouse tissue and embryonic stem cells. Read depth traces (left images) and Hi-C contact maps are as described in Fig 3.

In contrast, the larger deletions in the mouse strains were all detectable by differential ATAC- and/or ChIP-Seq analysis (Fig 4). The 1 Kb Kcnc3 deletion resulted in the loss of an H3K36me3 ChIP-Seq peak. The ~13 Kb Sox2 deletion resulted in the loss of ATAC-Seq, H3K27Ac ChIP-Seq, and H3K36me3 ChIP-Seq peaks, and the ~40 Kb SE15 deletion resulted in the loss of ATAC-Seq and H3K27Ac peaks. Of the mouse strains, only the Sox2 mutant had a tertiary structural change at the primary deletion site, which was the loss of the encompassing loop. The Sox2 mutant was also the only sample to have a significant change in a secondary structure (H3K27Ac modification) proximal to the primary site (Fig 4). All other differential peaks were contained within the boundaries of the deleted regions. The larger deletions in the Sox2 and SE15 mutants are visible in the Hi-C contact maps (Fig 4).

### Identification of secondary mutations

**Fig 5:**
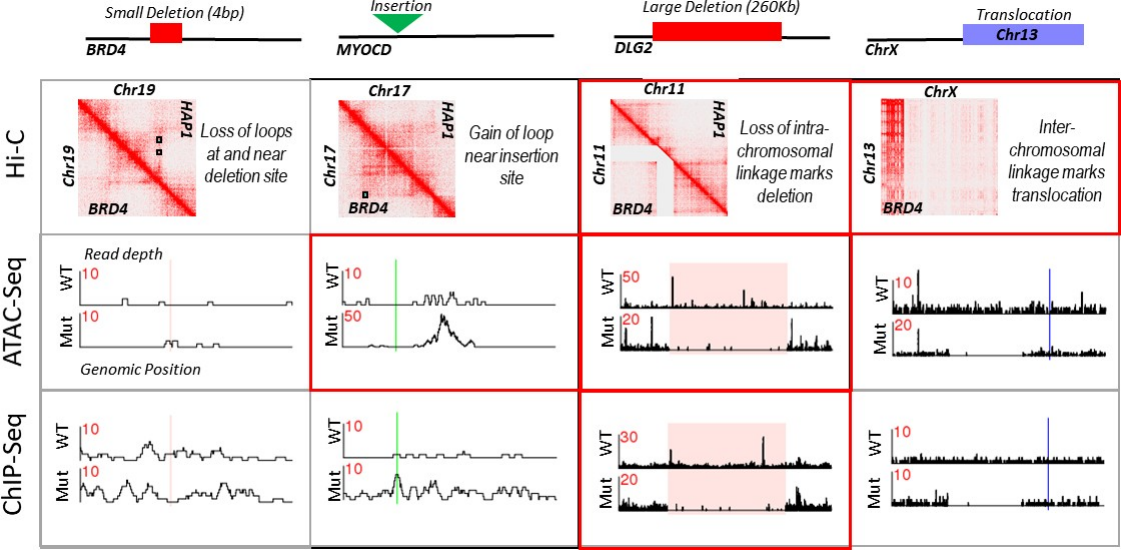
Summary of mutation types identified by multi-level structural analysis. Impact of the four different mutation types that were agnostically identified through our holistic analysis on secondary and tertiary DNA structures. All four mutations are found in the BRD4 mutant. The name listed under the mutation type is the gene or chromosome impacted by this mutation. ATAC- and ChIP-Seq read depth traces are as described in Fig 3. Hi-C contact maps for the left three mutation types show data for the modified strain in the lower left portion of the heat map, and for the un-modified strain in the upper right portion. Hi-C contact data for the translocation is only shown for the mutant strain. Black boxes designate loops called for each respective data sets. Colored boxes on the read traces designate the deleted region (red), insertion site (green), or putative chromosomal breakpoint (blue). Boxes that have a red border designate platforms where there structural change was significantly different as determined by differential analysis. ChIP-Seq data is for the H3K27Ac antibody only. Expanded analyses for these mutations are given in Fig 3, S5 Fig, S3 Fig, and S6 Fig.

To determine whether we could identify secondary mutations (i.e., mutations not at the directly targeted site) using multi-level DNA structural data, we performed a comprehensive analysis on a single modified HAP1 strain, BRD4. We were able to agnostically identify four different mutation types (small indels, insertion, large deletion, and chromosomal rearrangement) in the BRD4 mutant as described below (Fig 5). To identify short indels (≤ 60 bp) and small nucleotide variations (SNVs) in the cumulative primary sequencing data from all platforms, we used FreeBayes [40] as described in Materials and Methods. The BRD4 mutant contained 358 short indels and 1,240 SNVs that were neither present in the parent strain nor in the additional modified cell lines in this study. These numbers were similar to those observed for the TOP2B (329 indels and 1,185 SNVs) and SMARCA4 (380 indels and 1,348 SNVs) mutant samples. The parent HAP1 strain only contained one indel and four SNVs that were not observed in any of its derived mutant strains. To assess the potential source of the additional mutations observed in the modified cell lines, we determined the similarity between the sequences surrounding each identified mutation and the 20 bp gRNA used to modify that strain. Only one mutation for each modified strain (the targeted deletion sites) had ≥ 15 bp homology to the gRNA sequence and were proximal to a PAM site of “NGG", suggesting these changes have arisen through a non-Cas9 mediated mechanism (i.e., genetic drift during cell culturing) [41].

An advantage of Hi-C is the identification of gross chromosomal rearrangements in chromosomal-contact maps. The BRD4 mutant contained a *de novo* translocation, where the first 32 Mb of Chromosome X was fused to Chromosome 13 (Fig 5, S3A Fig). There was a 15 bp match to the 20 bp gRNA downstream of the breakpoint on Chromosome X (Fig 5, S3 Fig), with a perfect match to the last 5 bps of the gRNA, which are thought to be the most important for CRISPR/Cas9 binding specificity [42]; although there was not a canonical PAM sequence of “NGG" next to the match. The region containing the putative break site could not be amplified by flanking PCR primers in the BRD4 strain, but could be amplified in all other HAP1 strains, confirming the breakage of Chromosome X was present only in the BRD4 mutant. Two translocations were observed for the Sox2 mutant: a fusion of Chromosome 13 to Chromosome 8 and a fusion of Chromosome 17 to Chromosome 14, with 15 bp matches to gRNA that are proximal to the suspected breakpoints on Chromosomes 8 and 14 (S3B Fig).

To guide our search for additional mutations, we focused on the intersection of differential signals between platforms (Fig 2). In the BRD4 mutant, differential ATAC-Seq, H3K27Ac ChIP-Seq, and Hi-C interaction analysis all pointed to a ∼260 Kb deletion on Chromosome 11 (S5 Fig) that was not present in any other strain. Two 15 bp matches to the gRNA sequence were present within and upstream to the deletion, but these matches were not proximal to a PAM site. Similarly, differential analysis for all platforms pointed to a new ∼280 Kb deletion on Chromosome 10 in the TOP2B mutant. Six, 15 bp matches to the gRNA were contained within this deletion, but again none were proximal to a PAM site (S5 Fig). Both deletions were confirmed through targeted PCR and sequencing (see Materials and Methods). We did not identify any new deletions by looking at intersectional analysis in the mouse samples.

The analysis described above primarily focused on differential peaks that showed a significant reduction in read depth in the mutant relative to the parent strain, which is indicative of a deletion. We were also interested in finding out whether a significant increase in read depth in the mutant strain was also indicative of a genomic modification. To test this, we PCR-amplified and sequenced the areas containing several significantly up-regulated differential ATAC-Seq peak in the BRD4 mutant. Surprisingly, we identified the incorporation of a mammalian expression vector commonly used in genome engineering into the host genome immediately upstream of one of these peaks (S6 Fig). Since ATAC-Seq measures changes in DNA accessibility, this significant increase in accessibility likely reflects the high transcriptional activity of the incorporated expression vector. To determine if this exogenous insertion could be detected *de novo* from the sequencing reads, we assembled all unmapped reads from the NGS data using velvet and used blastn to determine which non-human sequences were among these assemblages (see Materials and Methods). Sequences that matched a different portion of the same expression construct were identified by this method, but their location in the genome could not be inferred directly just from the assembled reads, illustrating the advantage of utilizing structural data to agnostically identify insertion sites.

## Discussion

Here, we describe how multi-level DNA structural analysis can be leveraged to identify mutations in the genome that range in scale from SNVs to small (bp) and large (Kb) indels and chromosomal rearrangements (Mb) (Fig 5). While mutation detection from whole-genome sequencing data is effective for small mutations (≤ 10 bp), large deletions and chromosomal structural changes can be difficult to identify from short-read sequencing data because of challenges in accurately aligning sequences that vary from the reference genome [29]. The use of differential structural analysis to identify these larger changes is successful because it effectively bins short read data into larger structural units for comparison. By searching for mutations in different levels of DNA organization, we gain a comprehensive view of the genome at multiple resolutions that guide search efforts and improve confidence in called mutations through cross-validating evidence from independent platforms.

Based on the limited number of samples in this study, DNA organization structures appear to be relatively insensitive to short genomic changes; for example, of the 1,598 unique SNVs and small indels identified in the BRD4 mutant, 109 were contained within called peaks, and only 4 of these peaks were identified as being significantly different between BRD4 and the HAP1 parent strain. It is unknown if these significant variations in peak densities were the direct result of these mutations, but the majority of peaks containing small mutations were not significantly different between the BRD4 mutant and the HAP1 parent strain. Notably, this insensitivity was observed for the targeted 4 bp and 41 bp deletions in the BRD4 and SMARCA4 mutants respectively, which are contained within H3K36me3 peaks, and exhibited no discernible effect on the presence of the H3K36me3 mark to the encompassing regions (Fig 3). The three CRISPR/Cas9-targeted deletions ≥ 1,000 bp (Fig 4) and the two newly identified large deletions (S5 Fig) resulted in significant changes to ATAC-Seq and/or ChIP-Seq peaks that overlapped these regions as expected. Structural elements proximal to the deleted regions appeared to be robust to neighboring large deletions. The targeted deletion site to the Sox2 super enhancer was the only deletion that significantly affected a nearby peak (Fig 4). Nucleosome shifts may occur in the regions flanking these mutations, but the resolution of our ChIP- and ATAC-Seq data did not allow us to accurately call nucleosome boundaries. However, this is possible using ATAC-Seq data sequenced at a high depth [43], and can be explored in future studies.

When we applied this approach to identify genomic alterations in the six modified mammalian samples, we were able to identify six large secondary mutations (chromosomal rearrangements and deletions ≥ 200 Kb) in four of the modified samples. Similarities between proximal sequences and the gRNA used to modify that strain suggest they may be CRISPR/Cas9- induced. This finding is consistent with the observation by Kosicki et al., that CRISPR/Cas9 often induces large genomic structural changes at targeted editing sites [44]. However, as all of these cell lines were obtained from commercial vendors, where detailed information about culturing time post-editing was not available, and which are more prone to spontaneous mutations [45–48], we cannot conclusively attribute these mutations to off-target CRISPR/Cas9 binding, cell culturing, or genomic instability caused by the targeted deletions in these strains. Furthermore, CRISPR/Cas9 efficacy has been shown to be higher in cells with an impaired DNA damage response, suggesting these compromised cells may be artificially selected for when generating CRISPR/Cas9 mutants [21–22], which could also lead to a higher rate of mutations in subsequent culturing. Regardless of the origin of the large structural changes identified in our analysis, these mutations were reliably detected using analysis of variation in different levels of DNA organization. We suggest that the approach described here offers a significant improvement over standard WGS for the identification of genomic and epigenomic perturbations as it facilitates the discovery of these large changes, while still capturing nearly whole genome sequencing data.

Each of the NGS-based methods used here have unique advantages for mutation detection. Hi-C proved to be the most powerful, as it provides nearly whole-genome sequencing data while facilitating the detection of large structural changes which can be difficult to identify with short-read data alone. Dixon *et al*. recently demonstrated that structural variants in cancer genomes can be reliably detected using Hi-C analysis with as little as 1X coverage [49], attesting to the mutation detection power of this method. While we observed added value for using ATAC-Seq and ChIP-Seq data for mutation detection here, these assays alone do not provide sufficient genomic coverage to capture all potential mutations (S1 Fig). As CRISPR/Cas9 binding is dependent on DNA accessibility [42], the use of these platforms enrich for those areas of the genome most likely to be modified by Cas9, and provide valuable information to enhance mutation discovery from higher genomic coverage data sets like Hi-C. ATAC-Seq and ChIP-Seq also provide information on the functional state of the genome, which proved useful in the identification of inserted expression elements (S6 Fig). These expression elements are commonly used in genetic engineering [50], and this approach can be applied to identify the undisclosed insertion of these elements for commercial, biomedical, or biodefense purposes. A limitation of a multidimensional DNA analysis approach is the increased cost and labor necessary to generate these data sets over WGS. The commercialization of ATAC-Seq, ChIP-Seq, and Hi-C has reduced the time and cost needed to perform these assays, making them feasible alternatives to WGS. The integration of these platforms into a single assay would further reduce costs and enable unbiased, agnostic detection of genome editing for use in the field, clinic or research laboratories.

## Materials and methods

### Modified and control samples

The CRISPR/Cas9 modified HAP1 cell lines and the HAP1 parent strain were obtained from Horizon Discovery Group plc as follows: BRD4 mutant strain (catalog number: HZGHC000937c007); TOP2B mutant strain (catalog number: HZGHC003697c011); SMARCA4 mutant strain (catalog number: HZGHC000922c004); and HAP1 parent strain (catalog number: C631). The gRNAs for generating the mutant HAP1 strains were selected through an in-silico process that calculates the likelihood of potential off-targets containing up to five base mismatches. Out of these off-targets a score per gRNA was calculated that considers the number of mismatches, location of mismatch on the gRNA sequence [51] and the distance between mismatches on the gRNA. The guide RNAs with the best scores are manually chosen for the knockout generation. Targeted deletions were validated through targeted PCR followed by Sanger sequencing. The modified and parental HAP1 cells were cultured in Iscove’s Modified Dulbecco’s Medium (IMDM) with 10% FBS and 1% Pen/Step.

Mouse liver tissue samples were obtained from the Jackson Laboratory: Kcnc3 mutant strain (catalogue number 028540) and C57BL/6NJ background (catalogue number 005304). Mouse embryonic cell lines (F123, Sox2, and SE15) were obtained through the Ludwig Institute for Cancer Research Ltd in agreement with the Whitehead Institute for Biomedical Research. These strains are derived from the F123 parent strain [33], and the Sox2 strain is further described in [34]. Briefly, target gRNAs were designed to minimize off-targets using an online tool developed by the Feng Zhang group (MIT) and target deletions were validated through targeted PCR followed by Sanger sequencing. Mouse ESC lines were cultured in Dulbecco’s Modified Eagle’s Medium (DMEM) with 15% KnockOut Serum Replacement (ThermoFisher Sciences), 0.1mM non-essential amino acids (ThermoFisher Sciences), 1x Glutamax (ThermoFisher Sciences), 50 μM B-mercaptoethanol, and 100 U/ml LIF (Cell Guidance Systems). For the first two passages, cells were grown on mouse embryonic fibroblast (MTI-GlobalStem) feeder plates prepared on 0.2% gelatin-coated plates and cultured in DMEM with 10% FBS. Accutase was used as a more gentle method of passing the F123 mESC cells, and cells were split very densely at about 1:2 for the first passage and about 1:4 for subsequent passages.

### ATAC-Seq library preparation and data analysis

The ATAC-Seq was performed by Active Motif as described by Buenrostro et al. [32], with some modifications based on Corces et al. [52]. Briefly, cell pellets were resuspended in lysis buffer, pelleted, and tagmented using the enzyme and buffer provided in the Nextera Library Prep Kit (Illumina, Inc.). Tagmented DNA was then purified using the MinElute PCR purification kit (Qiagen), amplified with 10 cycles of PCR, and purified. The ATAC-Seq libraries were sequenced as 45 bp paired-end libraries on a NextSeq 500 and mapped to the human (version hg19) or mouse (version mm10) genomes using BWA [53] with default parameters. Reads were filtered using Illumina’s sequence quality filters, and PCR duplicates were removed. Peaks were called using MACS1.4.2 at a cutoff of p-value = 1e-7, without control file, and with the–nomodel option [54]. Peaks were merged for all samples of the same background, reads were counted for each region, and differential analysis was performed between each modified sample and its parent strain using DESeq2 [37].

### ChIP-Seq library preparation and data analysis

ChIP-Seq was performed by Active Motif using the following method. Mouse and human samples were fixed with 1% formaldehyde for 15 minutes and quenched with 0.125 M glycine. Cell lysates were sonicated and the DNA was sheared to an average length of 300–500 bp. Genomic DNA (Input) was prepared by treating aliquots of chromatin with RNase, proteinase K, and heat for de-crosslinking, followed by ethanol precipitation. An aliquot of chromatin (30 μg) was precleared with protein A agarose beads (Invitrogen). Genomic DNA regions of interest were isolated using 4 μg of antibody against the histone modifications H3K27Ac and H3K36me3. Complexes were washed, eluted from the beads with SDS buffer, and subjected to RNase and proteinase K treatment. Crosslinks were reversed by incubation overnight at 65°C, and ChIP DNA was purified by phenol-chloroform extraction and ethanol precipitation.

Illumina sequencing libraries were prepared from the ChIP and Input DNAs by the standard enzymatic steps of end-polishing, dA-addition, and adapter ligation. After a final PCR amplification step, the resulting DNA libraries were quantified and sequenced on Illumina’s NextSeq 500 (75 bp single end reads). These sequencing reads were mapped to the human (version hg19) or mouse (version mm10) genomes using BWA [53] with default parameters. Reads were filtered using Illumina’s sequence quality filters, and PCR duplicates were removed. Peaks were called using MACS2 with the parameters ‘-f BAM -g hs -s 36 -–nolambda -–nomodel’ [54]. Non-immunoprecipitated chromatin input libraries were also generated and sequenced for each strain and used to improve peak calling accuracy by MACS2.

Differential peak analysis was performed for each mutated sample relative to the unmodified parent strain using DESeq2 [37]. Called peaks were not merged prior to differential analysis as done with ATAC-Seq analysis to maximize the detection resolution. Instead, peaks for a single replicate for each sample were used to determine read counts in those regions for all other samples of the same background using the countRangeset function in the systemPipeR library for R [55]. Differential analysis was then performed on all peaks called for a single mutated sample relative to its parent strain. As some peaks present in the parent strain may have been lost in the modified strains, the same analysis was repeated for all called peaks for the parent strain. Differential peaks identified using the mutated and parent strain peak sets were combined and overlapping peaks in this set were then merged. Read counts were subsequently determined for these merged peaks for the mutated and parent strains, and differential analysis was repeated.

Venn diagrams that show the overlap in differential peaks across platforms (Fig 2) were generated using the VennDiagram library for R. Peaks from two platforms were counted as overlapping if they were within 5 Kb from each other. As multiple peaks for one platform can overlap the same peak in a different platform, overlapping peak counts can vary depending on which platform is used as the reference. For each platform comparison, we used the platform with the smaller number of peaks as the reference. Overlap data for each called differential peak for each platform are reported in S1–S6 Tables.

### Hi-C library preparation and data analysis

Hi-C data were generated using a pre-commercial version of the Arima-Hi-C technology, whereby only a single restriction enzyme (as opposed to multiple) is used for chromatin digestion. To prepare mouse tissue samples for Hi-C analysis, tissues were harvested and snap-frozen, pulverized, and crosslinked as previously described [56]. Then, tissue was further dissociated and nuclei were isolated as described previously [57]. The resulting nuclei were input into the Hi-C protocol. For cell lines, 1 million crosslinked cells were used as input into the Hi-C protocol. Briefly, chromatin from crosslinked cells or nuclei was solubilized, and then digested using a single restriction enzyme recognizing the GATC motif. The digested ends were then labeled using a biotinylated nucleotide, and ends were ligated to create ligation products. Ligation products were purified using solid phase reversible immobilization (SPRI) magnetic beads, fragmented, and size-selected using SPRI beads. Biotinylated fragments were then enriched using streptavidin beads, and Illumina-compatible sequencing libraries were constructed on-bead using a modified workflow of the KAPA Biosystems, Inc.’s Hyper Prep kit. The bead-bound library was then amplified, and amplicons were purified using SPRI beads and subject to deep sequencing.

Hi-C data pre-processing was conducted using Juicer [58]. Human data were aligned to the hg19 reference genome, and mouse data were aligned to the mm10 reference genome. After pre-processing and generating .hic files using Juicer Pre, loops were called using HICCUPS [8] and TADs were identified using the “Arrowhead" algorithm within the Juicer tool. Hi-C data were visualized using Juicebox [59] with Balanced normalization applied to the matrices. For differential Hi-C analysis we used the diffHiC software [39], a tool that models biological variability between biological replicates to test for statistically significantly differential interactions between two biological conditions. Because diffHiC contains its own data normalization strategy, raw Hi-C contact matrices were generated at 25 Kb resolution using Juicebox “dump". These matrices were re-formatted and then matrices from biological replicates of Condition 1 and matrices from biological replicates of Condition 2 were input into diffHiC to conduct two types of analyses: differential interaction analysis and differential “peak" analysis. After data normalization and filtering low abundance bin-pairs, all differential interactions were identified genome-wide and are herein referred to as Differential Interactions. For differential loop (or “peak") analysis, the ‘filterPeaks’ function was used to identify peaks and then these peak positions were compared across conditions. For both differential Hi-C analyses as well as False Discovery Rate (FDR) calculations, a cutoff of 0.05 was used to define statistical significance.

### PCR verification and identification of exogenous insertions

PCR primers were designed to amplify the regions surrounding differential peaks, putative deletion boundaries, or putative chromosomal break points. Following PCR, the amplified regions were visualized on an agarose gel to confirm expected fragment size, and then select samples were sequenced by Sanger Sequencing. A list of all PCR primers is provided in S7 Table. To identify the presence of exogenous insertions: unmapped reads were identified from BWA generated BAM files using samtools view -b -f 4 [53], assembled using velvetg with the options -cov_cutoff 5.2 [60], and aligned to all publicly available sequences using blastn [61].

## Supporting information

S1 Fig Genomic coverage of each platform. The number of sequencing reads at each base (fold coverage) was determined for each platform individually and cumulatively. Here, we show the fraction of the genome (y-axis) that has at least some specified depth of sequencing coverage (x-axis). The key in the right corner designates which curve corresponds to which platform.
(TIF)

S2 Fig Peak densities are correlated between modified and parent strains. The number of reads within each called peak was plotted for the reference strain (x-axis) against the CRISPR/Cas9-edited strain (y-axis). We show a representative plot for each data type and background strain used. A fit regression line and corresponding R^2^ are shown in red.
(TIF)

S3 Fig Unique translocations present in modified samples. (A) The top image is an all chromosome by all chromosome Hi-C contact map for the BRD4 mutant (bottom/left half) and the HAP1 parent strain (top/right half). Each row and column represents a single chromosome. For simplicity, we only labeled those chromosomes where there was a difference between the mutant and parent strain, which is marked with a gray box. The lower image is an enlargement of the above boxed region to show the extent of inter-chromosomal linkage in the mutated sample. Sequences near the putative breakage point that are homologous to gRNA sequences are shown below. Red bases signify match to gRNA at that position. (B) is the same as (A) except for the Sox2 mutant which had two new translocations relative to its parent strain.
(TIF)

S4 Fig Unique intra-chromosomal rearrangements present in a modified sample. Hi-C contact map for Chromosome 15 for the SE15 mutant (left) and the F123 parent strain (right). The sites of intra-chromosomal rearrangements specific to the SE15 mutation are marked with black boxes.
(TIF)

S5 Fig Large deletions identified in modified HAP1 cell lines. Read depth traces (left images) and Hi-C contact maps (right images) are as described in Fig 3. Dashed lines mark the center of large deletions in Hi-C contact maps. Vertical blue line in the “Gene Annotation" track signify location of sequences with ≥ 15 bp homology to the gRNA sequence.
(TIF)

S6 Fig Identification of inserted expression vector through altered DNA accessibility. (A) Read depth traces (left images) and Hi-C contact map are as described in Fig 3. Dashed lines mark the insertion location in the Hi-C contact map. (B) Blue arrows below the read traces mark the region of the genome amplified by PCR and sequenced. These sequencing data identified the insertion of a mammalian expression plasmid pFB-Neo-E-hA20 immediately upstream of the differential ATAC-Seq peak. (C) Alignment of inserted sequence identified by targeted PCR of ATAC-Seq peak (green bar) and contigs assembled from unaligned sequencing reads (red bars) to the pFB-Neo-E-hA20 expression plasmid.
(TIF)

S1 Table Summary of differential analysis for all platforms for BRD4 mutant. Each tab contains all significantly different regions between BRD4 and its parent strain for each data type. Tab 1 is for H3K27Ac ChIP-Seq data; Tab 2 is for H3K26me3 ChIP-Seq data; Tab 3 is for ATAC-Seq data; Tab 4 is for Differential Hi-C interaction analysis; and Tab 5 is for Differential Hi-C peak analysis. The last 4 columns for each Tab reports the number of differential regions for the indicated platform that overlapped or was within 5 Kb of that called region. For Tabs 1–3, each column corresponds to the following: chr—chromosome containing differential peak; start- peak start coordinate; stop—peak stop coordinate; meanXXX is the average read density for that peak in that strain; pval—calculated p-value; padj—adjusted p-value as determined by DESeq2. For Tabs 4–5, two sets of coordinates are given (chrom, start, end) to designate genomic regions that are interacting. Differential Hi-C analysis was performed using diffHiC, which reports FDR instead of adjusted p-value. S1–S6 Tables have the same format.
(XLS)

S2 Table Summary of differential analysis for all platforms for TOP2B mutant. (XLS)

S3 Table Summary of differential analysis for all platforms for SMARCA4 mutant. (XLS)

S4 Table Summary of differential analysis for all platforms for Kcnc3 mutant. (XLS)

S5 Table Summary of differential analysis for all platforms for Sox2 mutant. (XLS)

S6 Table Summary of differential analysis for all platforms for SE15 mutant. (XLS)

S7 Table PCR primers used to validate identified mutations. (XLS)

S8 Table Summary of the number of biological replicates per sequencing platform for each sample. (XLSX)
