# Supplementary figures and images for: Agnostic detection of genomic alterations by holistic DNA structural interrogation
Source: PLoS One. 2018 Nov 29;13(11):e0208054. doi: 10.1371/journal.pone.0208054 (PMC6264503; doi:10.1371/journal.pone.0208054)

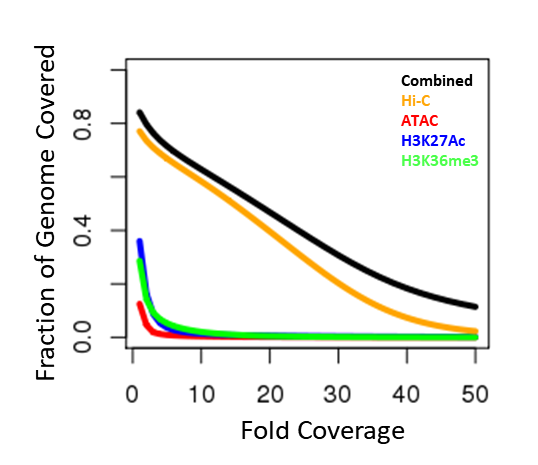

Supplement: S1 Fig — The number of sequencing reads at each base (fold coverage) was determined for each platform individually and cumulatively. Here, we show the fraction of the genome (y-axis) that has at least some specified depth of sequencing coverage (x-axis). The key in the right corner designates which curve corresponds to which platform. (TIF) [file pone.0208054.s001.tif]

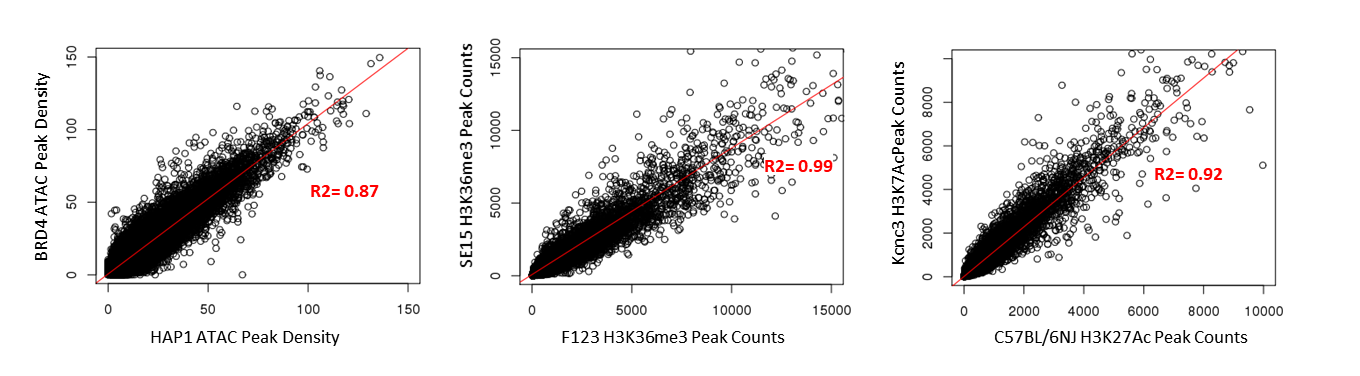

Supplement: S2 Fig — The number of reads within each called peak was plotted for the reference strain (x-axis) against the CRISPR/Cas9-edited strain (y-axis). We show a representative plot for each data type and background strain used. A fit regression line and corresponding R2 are shown in red. (TIF) [file pone.0208054.s002.tif]

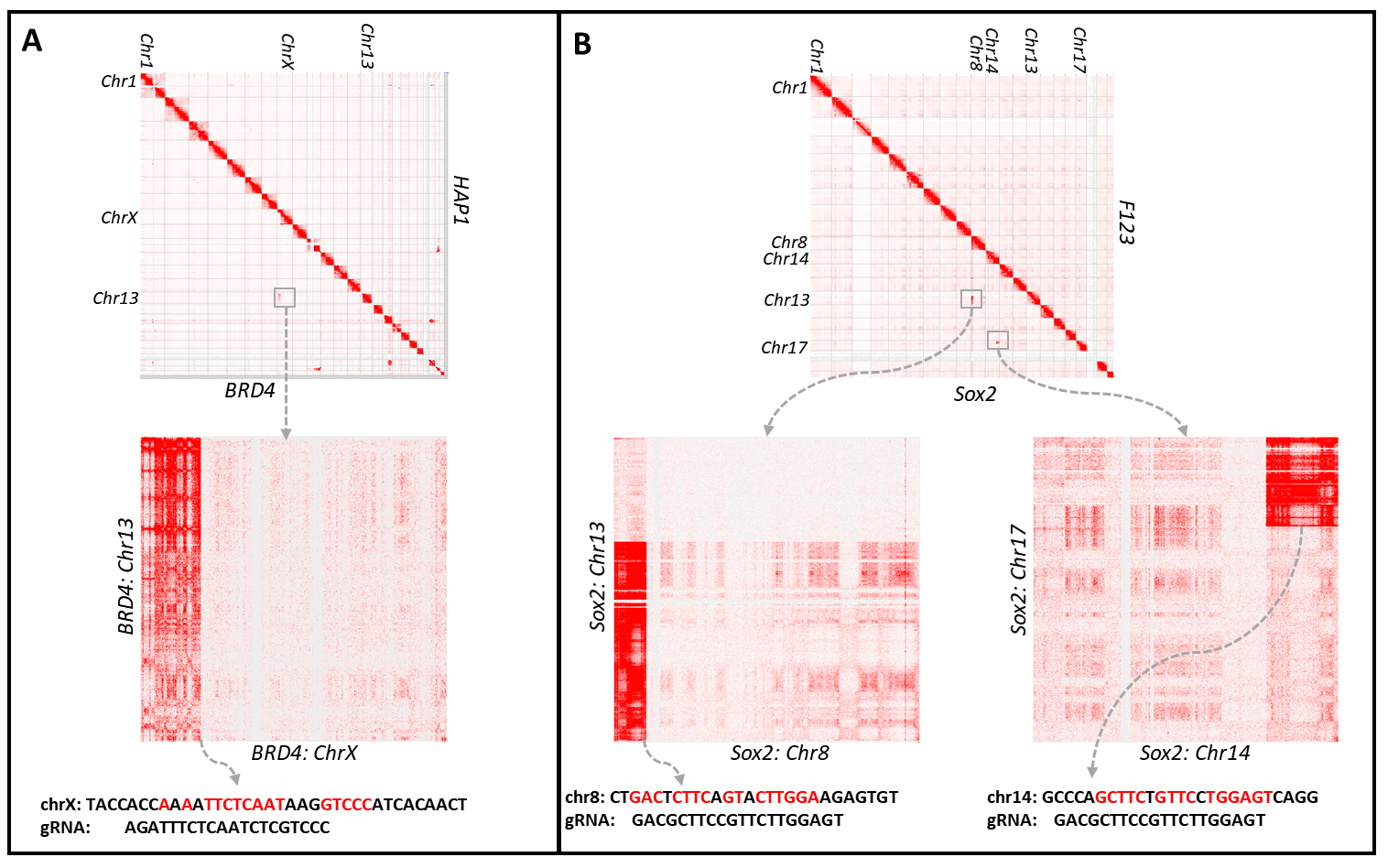

Supplement: S3 Fig — (A) The top image is an all chromosome by all chromosome Hi-C contact map for the BRD4 mutant (bottom/left half) and the HAP1 parent strain (top/right half). Each row and column represents a single chromosome. For simplicity, we only labeled those chromosomes where there was a difference between the mutant and parent strain, which is marked with a gray box. The lower image is an enlargement of the above boxed region to show the extent of inter-chromosomal linkage in the mutated sample. Sequences near the putative breakage point that are homologous to gRNA sequences are shown below. Red bases signify match to gRNA at that position. (B) is the same as (A) except for the Sox2 mutant which had two new translocations relative to its parent strain. (TIF) [file pone.0208054.s003.tif]

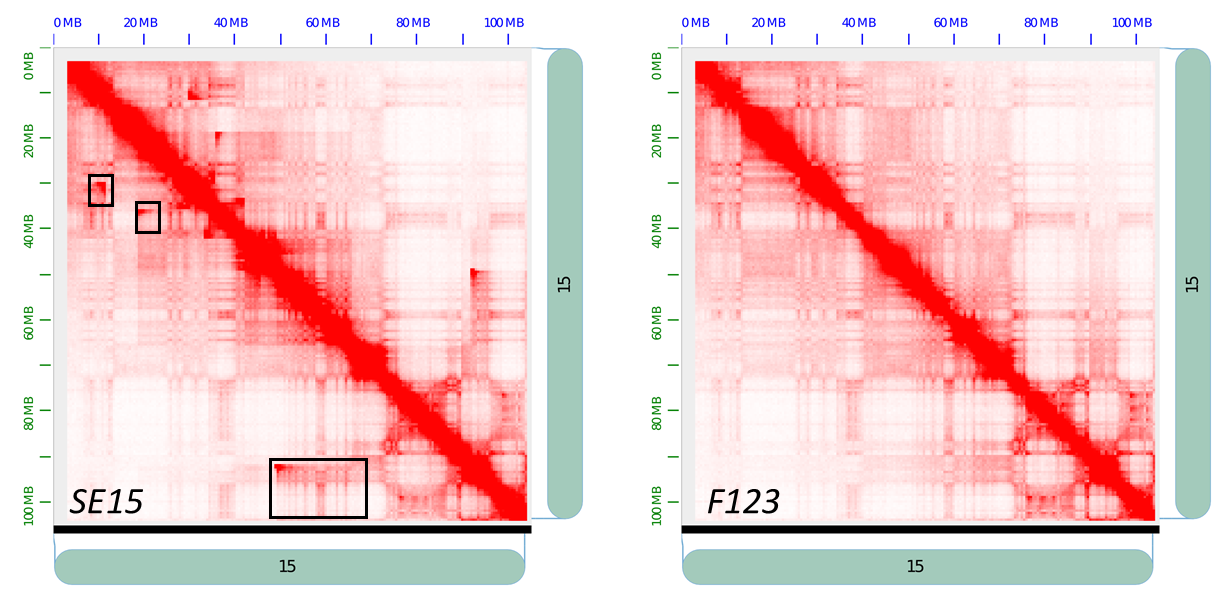

Supplement: S4 Fig — Hi-C contact map for Chromosome 15 for the SE15 mutant (left) and the F123 parent strain (right). The sites of intra-chromosomal rearrangements specific to the SE15 mutation are marked with black boxes. (TIF) [file pone.0208054.s004.tif]

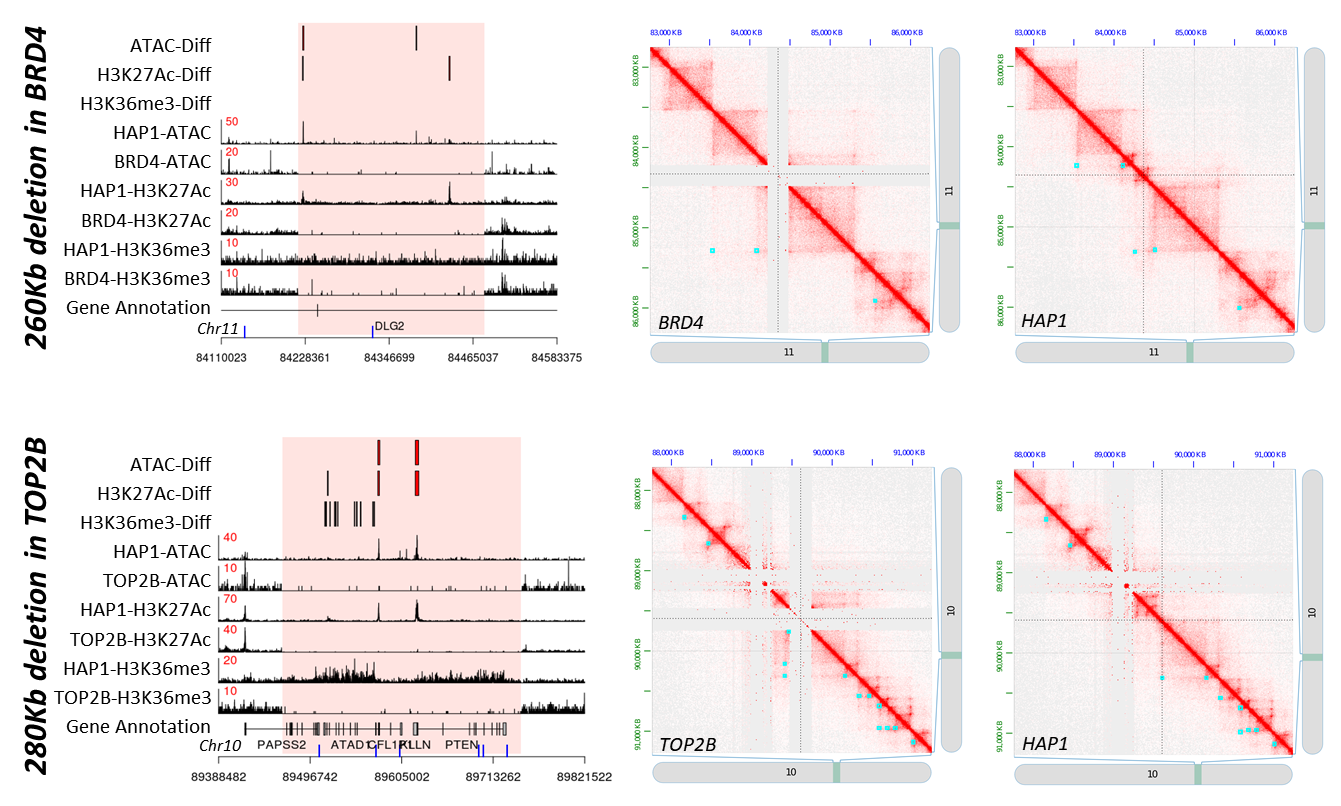

Supplement: S5 Fig — Read depth traces (left images) and Hi-C contact maps (right images) are as described in Fig 3. Dashed lines mark the center of large deletions in Hi-C contact maps. Vertical blue line in the “Gene Annotation" track signify location of sequences with ≥ 15 bp homology to the gRNA sequence. (TIF) [file pone.0208054.s005.tif]

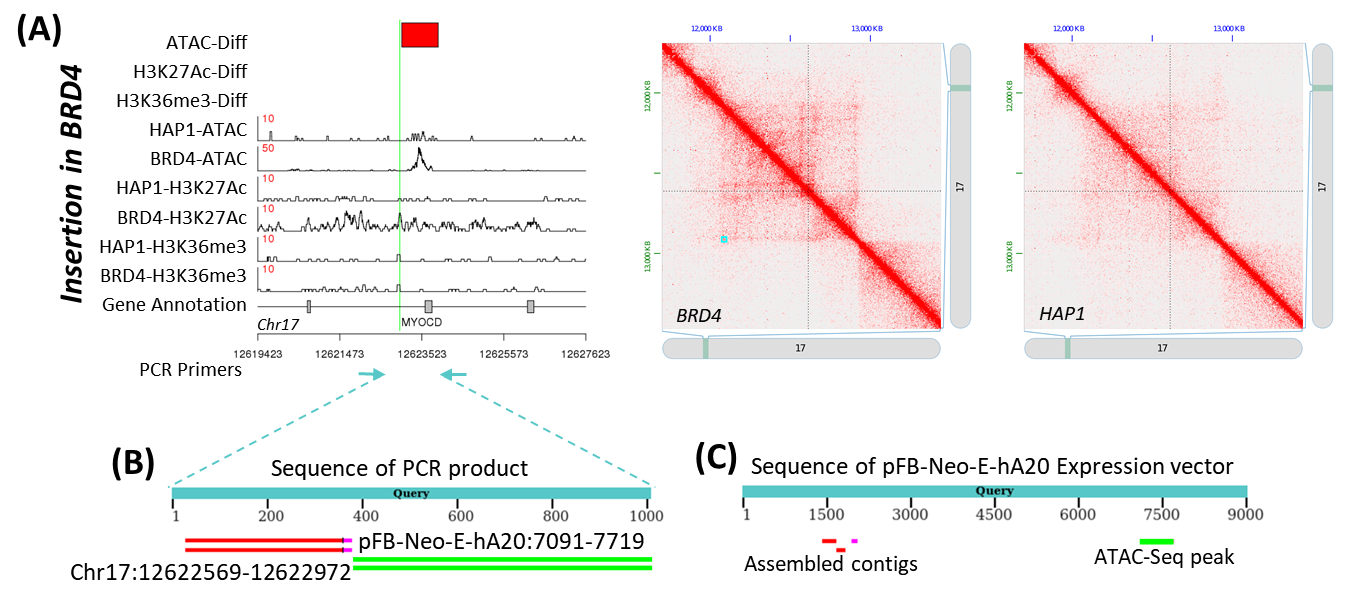

Supplement: S6 Fig — (A) Read depth traces (left images) and Hi-C contact map are as described in Fig 3. Dashed lines mark the insertion location in the Hi-C contact map. (B) Blue arrows below the read traces mark the region of the genome amplified by PCR and sequenced. These sequencing data identified the insertion of a mammalian expression plasmid pFB-Neo-E-hA20 immediately upstream of the differential ATAC-Seq peak. (C) Alignment of inserted sequence identified by targeted PCR of ATAC-Seq peak (green bar) and contigs assembled from unaligned sequencing reads (red bars) to the pFB-Neo-E-hA20 expression plasmid. (TIF) [file pone.0208054.s006.tif]
